# The Influence of Accreditation on the Sustainability of Organizations with the Brazilian Accreditation Methodology

**DOI:** 10.1155/2018/1393585

**Published:** 2018-01-16

**Authors:** João Éderson Corrêa, João Batista Turrioni, Anderson Paulo de Paiva, Vinicius de Carvalho Paes, Pedro Paulo Balestrassi, Pedro José Papandrea, Ernany Daniel de Carvalho Gonçalves

**Affiliations:** ^1^Industrial Engineering and Management Institute, Federal University of Itajuba, 1303 BPS Avenue, 37500-903 Itajubá, MG, Brazil; ^2^Vilanova i la Geltrú School of Engineering, Universitat Politècnica de Catalunya, Víctor Balaguer Avenue, 08800 Vilanova i la Geltrú, Barcelona, Spain

## Abstract

This research evaluates the influence of the Brazilian accreditation methodology on the sustainability of the organizations. Critical factors for implementing accreditation were also examined, including measuring the relationships established between these factors in the organization sustainability. The present study was developed based on the survey methodology applied in the organizations accredited by ONA (National Accreditation Organization); 288 responses were received from the top level managers. The analysis of quantitative data of the measurement models was made with factorial analysis from principal components. The final model was evaluated from the confirmatory factorial analysis and structural equation modeling techniques. The results from the research are vital for the definition of factors that interfere in the accreditation processes, providing a better understanding for accredited organizations and for Brazilian accreditation.

## 1. Introduction

Currently, health managers are increasingly concerned with issues related to improving the quality of services provided and improving care processes. In this sense, quality management became paramount for hospitals to achieve their goals, generating results for patients and for the institutions. With an increasingly competitive market, several hospitals, public and private, are facing great challenges in their management. In view of these uncertainties, there is a need to seek solutions to improve quality and cost-effectiveness [1]. In this context, hospital accreditation appears as an approach for improving the quality of health services [2].

The management of accreditation quality and effectiveness has been a subject of great interest by researchers in the health care area, since reliable and consistent research is an important factor in the development of the accreditation process in organizations. The success in identifying factors that directly affect the quality management process and accreditation is seen as crucial for sustainable performance, given that “most health care managers” are seeking to adopt new strategies and management tools that enable organizations to be more competitive, meeting customer expectations, and avoid costs by reducing errors and waste and enabling improved organizational performance [3]. Several factors have contributed to the adoption of quality management strategies by the organizations in order to improve their performance and obtain greater market share [4].

Hospital accreditation has been prominent in health environments, as an important approach in the process of improving quality management; however, few studies have been published seeking to analyze the performance of organizations that have been using hospital accreditation [3, 5–9]. In Brazil, hospital accreditation began to be discussed in 1995, when the Ministry of Health created the program “Quality Assurance and Improvement in Health,” which led to the formation of a technical committee, responsible for the elaboration of the new quality guidelines in the country that resulted in the creation of the “Brazilian Manual of Hospital Accreditation” and the Brazilian Accreditation System (SBA).

The standard established by the Brazilian manual is based on three levels of complexity: level 1: accredited, refers to the existence of processes aimed at ensuring the safety of the patient; level 2: full credential, refers to the integrality of the management, involving the monitoring of security barriers, processes, and protocols implemented, involving a critical analysis of the controls of care processes, establishments of action plans, improvement plans, and the intersectoral interaction; level 3: accredited with excellence, the organization has already incorporated a critical follow-up of the designed processes of its assistance results, developing cycles of improvement in a systematic way; decision making aligned with institutional strategic planning [10].

For the implementation of this process, the National Accreditation Organization (ONA) was created, a private law entity, responsible for the development, application, and follow-up of standards for the accreditation process in Brazil, with the objective of creating mechanisms for improving health services. Currently, the Brazilian Accreditation Methodology created by ONA is internationally recognized by ISQua and can be used in health services around the world [10, 11].

### 1.1. Organization Profile

**Figure 1:**
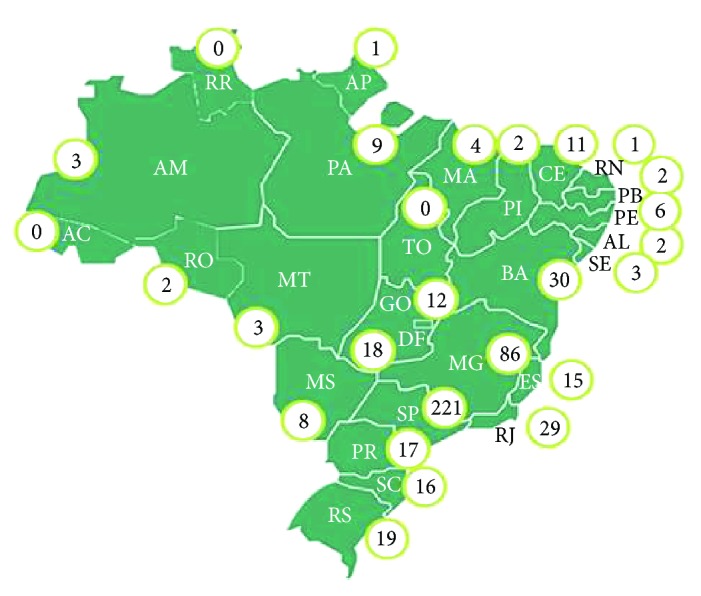
Map of the distribution of health services certified by ONA in Brazil. Source: ONA [12].

A search conducted in June 2016 in the ONA database identified that 515 health institutions are accredited by the organization.  Figure 1 shows the geographic location of the services accredited by ONA in Brazil.

However, when the geographical distribution of these institutions is verified in the Brazilian map, it is possible to observe a great inequality, being most of them located in the southeast region. This scenario shows that despite the establishment of the Brazilian Model of Hospital Accreditation, even after a decade of the implementation, it has not yet reached all regions as it should, highlighting the state of São Paulo with 221 health services, corresponding to 42.5% of accredited organizations throughout the country. According to the health service distribution ONA [12], it is possible to conclude that most hospitals in Brazil do not meet the requirements and quality standards required by the ONA. On the other hand, the increase in customer demands, market, and the public sector itself has forced institutions to meet the basic quality requirements, such as security and quality assurance of the services.

## 2. Literature Review

Quality management in health services has revealed that accreditation is a tool that contributes to competitiveness in the global health market; this approach was stimulated by impressive results in the industrial sector [1, 13]. Woldgebriel et al. [14] developed a study that evaluates the efforts made to improve health quality by reporting the use of different approaches, and the accreditation has been used on a large scale.

A study by the World Health Organization (WHO) in 2000 showed that hospital accreditation is directly influenced by technical competence, team integration, and personal motivation [15].

Claver et al. [3] and Xiong et al. [16] report that few scholars are willing to discuss, evaluate, or measure the results of hospital accreditation. On the other hand, theoretical models have been applied to guide health research studies and use the formal evaluation models such as the European Quality Award (EFQM), the Malcolm Baldrige National Quality Award (MBNQA), and the Deming Award [17, 18]. These models are used to examine the relationships between quality and accreditation constructs [1, 19–23]. The EFQM model has been widely used, becoming an efficient tool in the process of evaluating the relationship between hospital performance and accreditation in public and private organizations [3, 24, 25]. It is also common to find instruments of quality measurement that adopt the model developed by Donabedian [26], seeking to identify the relations between three factors: structure, process, and result [27, 28]. In this context, it can be observed that several studies have developed instruments for measuring the quality of health services.

### 2.1. Critical Factors That Influence Quality Management of Health Services

In order to measure the influence of quality management on the sustainability of organizations, it is necessary to identify the main factors that have affected the performance of accredited organizations, so an extensive research was developed in the literature that deals with this issue as an objective to identify these factors. From this research, it was possible to identify the main constructs that have been used to measure the effect of quality management with a focus on accreditation in organizational sustainability.  Table 1 summarizes the constructs found.

As seen in Table 1, the constructs used to measure the quality of health services are related to different dimensions, ranging from evaluation of administrative processes to clinical processes, in order to measure the results for the organization and patients.

## 3. Research Methodology

**Figure 2:**
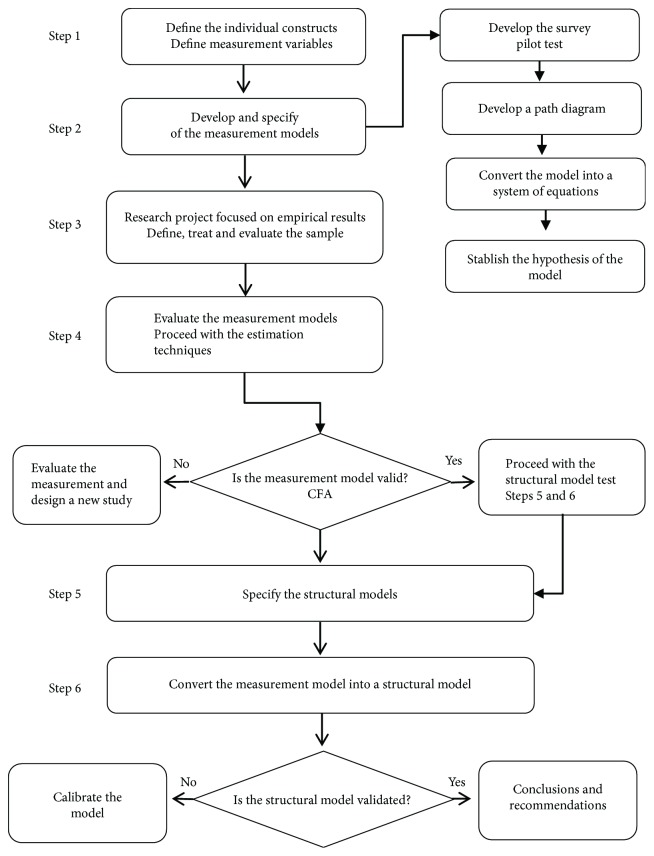
Research project roadmap. Source: adapted from Forza [43] and Hair et al. [44].

This research uses the survey method based on the work of Forza [43], which has been widely used for the development of research related to the evaluation of quality in health services, and strives to interpret the reality of organizations. However, some authors report that qualitative methods are limited by identifying only patterns of behavior, not searching deeper into causal relationships. In this sense, the use of structural equation modeling (SEM) techniques with the proposed method was combined.  Figure 2 shows the steps suggested by Hair et al. [44], adding the propositions established by Forza [43].

### 3.1. Step 1: Development of Operational Definitions

At this stage, it is important to have a good literature review, a necessary condition for the construction of a reliable model, and to obtain useful results in SEM; it also involves the prioritization of the constructs found in the literature, in order to substantiate and justify the objective of the current research [43, 44].

### 3.2. Step 2: Measurement Model Development

Once the latent constructs and their respective measurement variables were defined, the measurement instrument (survey) was developed using a Likert scale [44]. The pilot test was developed with the purpose of evaluating the operationalization of the constructs and the validity of the questions used, through the suggestions of the group of experts who contributed to the elaboration of the survey according to the propositions established by Forza [43]. After choosing the constructs as well as their respective measurement variables, a path diagram of the model was constructed using the Path analysis method [44, 45]. After the construction of the model through a path diagram, the model was transformed into a system of equations, since many software used in MEE require this construction. The construction of the system of equations was developed based on the techniques established by the SEM, which is a confirmation procedure and not very exploratory [44]. The model of measurement of the dependent or endogenous variables is represented by (1) and described in Table 2 [46, 47].

(1) $$\begin{array}{c} y=\Lambda_{y}\;\eta +\varepsilon . \end{array}$$

From (1), the equations of the measurement model for the constructs selected for this research were developed and are shown in Table 3.

Once the equations for the factors were defined, the research hypotheses were developed, which represent a series of hypothetical cause and effect relationships between the variables according to Table 4.

### 3.3. Step 3: Empirical Result Research

At this stage, questions regarding issues related to the research project were considered [48]. The data used in the research correspond to the data collected by a survey conducted among all institutions accredited by the Brazilian hospital accreditation methodology. In this stage, there were questions regarding location, type of organization (public, private), classification (profitable or philanthropic), number of employees, number of beds, and level of accreditation (level 1: accredited; level 2: full accreditation; and level 3: accredited with excellence). The data were collected through a survey from SurveyMonkey, through simple sampling for an initial sample of 515 accredited institutions selected to answer the survey. Of these, 49.51% (two hundred and fifty-five) completed the survey, being the sample to be evaluated. The size of the sample was considered adequate according to the propositions established by Kline [49], Hair et al. [44], Reinartz et al. [50], M. Hill and A. Hill [51], and Alwin [52]. Analyzing the collected sample, it was possible to verify that a good part of the surveys were answered by quality managers with nursing training, with a high predominance for employees who work in the organization more than four years, corresponding to 54.90% of the answers (164 answers). Almost seventy-eight percent (78.08%) of the organizations that participated in the research are located in the southeastern region of Brazil and offer 151 to 500 beds, and almost thirty percent (30.74%) belong to the group of public organizations and 69.26% private; from these results, 41.55% are philanthropic. These results are consistent with the distribution of accredited organizations in Brazil, and most of the accredited institutions are hospital type. Of the total responses obtained, 123 organizations are accredited with excellence, followed by 89 accredited organizations at the full level, 71 accredited, and 8 organizations have a certification seal. Steps 4, 5, and 6 will be presented in the following sections.

#### 3.3.1. Discussion on the Results Found

Before starting to evaluate the measurement models, some procedures were performed according to the propositions established by Anderson and Gerbing [53] and Vieira [54]. Initially, the exploratory factorial analysis was performed only as a data purification procedure, from a traditional perspective. Afterwards, a confirmatory factorial analysis was performed considering the following criteria: dimensionality reduction, convergent validity, reliability, and discriminant validity through LISREL®. However, before the above steps could be developed, it was necessary to make some procedural decisions that resulted in the following information: the software chosen for the analyses of this study was LISREL, using the data obtained from a covariance matrix, through of the estimation technique: maximum likelihood (ML), with the level of abstraction: partial aggregation.

No sum of scale problems was identified, and the indices chosen for model evaluation were based on the suggestions described by Baumgartner and Homburg [55, 56].

The data were adequately treated, and no coding errors were identified in the data collected [49]. The outlier analysis was done from a discriminant analysis, using the Mahalanobis distance to classify the observations in their predicted groups using Minitab® software.

The treatment of missing values was developed from the complete case study method (Listwise) established by Hair [44], and the reliability of the sample was proven from the results obtained from the Cronbach's alpha coefficient, which presented values higher than 0.8. Observations were considered independent since the surveys were answered by different individuals and institutions, providing simple random sampling. The linearity of all relationships was verified from the analysis of sample covariance, where no null covariances were identified. From the results obtained for the asymmetry test (−1.956) and kurtosis (4.750), the Mardia coefficient (22.615) did not indicate severe violations of the normality assumption.

The absence of the multicollinearity phenomenon was confirmed by the results of the determinants > 0.00001 of the correlation matrix, calculated in the LISREL software. These results can be verified in Table 5.

The absence of multicollinearity was confirmed by the results of the variance inflation factor (VIF) < 5, due to the nonexistence of correlations above 0.8 and by *R*^2^ values of 0.54 [44, 47].

The principal component analysis method was chosen [53]. In order to perform the factor analysis, the free parameters (=108) and the number of variables observed from the formula (*v* (*v* + 1)/2) were identified—resulting in the value 820. Based on the calculated values, the model of confirmatory factorial analysis obtained 712 degrees of freedom (820–108). In this case, the results of the modification indexes for the model indicated an adequate adjustment for the data collected [44, 57].

The evaluation of the measurement models for the constructs (L, PM, O, Q, P, S, A, and SY) was developed according to the propositions established by Vieira [54]. The Bartlett's sphericity test results were very small (*p* ≤ 0.001), while the results obtained by the Kaiser-Meyer-Olkin test (KMO > 0.8) presented satisfactory results for all evaluated constructs.

### 3.4. Step 4: Measurement Model Evaluation

The results of the descriptive statistics developed using the technique of factor extraction by the principal component method with the nonrotated solution developed from the analysis of the covariance matrix can be verified in Table 6.

Analyzing the results obtained from the method of factorial analysis for the selected constructs, it can be verified that most of the constructs can be represented by two factors, except for the constructs accreditation and process orientation represented by only one factor and sustainability construct represented by three factors. All factors present commonality results above 75% of explanation, which are considered acceptable [44, 49, 54].

The factors related to the leadership construct, leadership performance nominations, and leadership involvement correspond with the factors from Mcfadden et al. [31], Awuor and Kinuthia [6], Lee et al. [21], and Woo et al. [30]. Similar results were identified in the literature for the other constructs. According to the analysis developed, it can be seen that the relationships between the variables are consistent with the perspectives found in the literature, and, in general, the factors represent the content of the questions that measure their respective construct.

### 3.5. Step 5: Structural Model Specification

**Figure 3:**
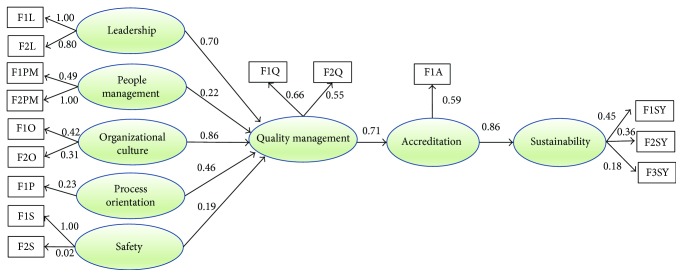
Results of structural modeling analysis.

After analyzing the measurement model of the latent variables, the results were grouped in a specification model; this model was estimated by the maximum likelihood method. The confirmatory factorial analysis was developed through the software LISREL version 9.2, in order to verify if the data fit a model. The relationships between variables, observed (independent) and latent (dependent), are illustrated in Figure 3.

This analysis can be done from the signals obtained for the respective estimated parameters; another form of verification is from the forces of these hypothetical links, which must be significant, that is, the *t* values must be greater than |1.96|. It is also necessary to verify the result of the variance that can be evaluated from the results obtained through the multiple square correlations (*R*^2^) for the structural equations [58]. From this result, it can be verified that some estimates are not within the normally accepted standards, and in terms of global adjustments, it can be said that the indexes of goodness of fit of the model correspond to the normally accepted limits; these results can be verified in Table 7.

From the results of the normed fit indices (NFI > 0.9), the index of comparative fit (ICF > 0.9), the relative fit index (RFI > 0.9), the goodness-of-fit index (GFI > 0.8) (PGFI < 0.5), and the standardized mean square error (RMSEA > 0.3 < 0.6), and absolute values of *R*^2^ > 2.58 in the standardized residue matrix, it can be confirmed that the data do not show potential threats to unidimensionality.

From the results obtained for the coefficient *γ* > 0.5, the standardized solution, and the *t* value, it can be stated that the models have sufficient evidence for the convergent validity [59]. The reliability of the constructs is proven from the value of Cronbach's alpha > 0.84; analyzing the results of the correlation between the constructs, it can be verified that the correlation between the variables did not exceed 0.7, suggesting the evidence of discriminant validity according to Steenkamp and Van Trijp [60]. Another test developed was the analysis of mean variance, where it can be observed that the results are greater than 0.50 [61]. So, in this case, the sustainability is explained by a set of relationships established between the other constructs. In general, it is possible to conclude that the interaction of exogenous variables (leadership, people management, organizational culture, guiding processes, and security) with an endogenous variable, like quality management, results in a relevant influence (high positive estimates) in the endogenous variable of accreditation that consequently generates a great influence (86%) on the sustainability of health organizations accredited by the methodology developed by ONA. These relationships can be confirmed in the literature, agreeing with the studies that have been developed in several countries; however, it has not been found in the literature studies that deal specifically with this type of relationship.

## 4. Power Assessment

In this step, the propositions established by Diamantopoulos and Siguaw [58] consider that the evaluation of power is important but often neglected in the process of evaluation of structural models.

This is the individual evaluation of the chi-square test, which is obtained from type I errors. In this case, a rejection of a correct model with type II error can occur. Complementarily, the power test is important due to the influence of sample size, since large samples tend to obtain various types of specification errors. Then, an analysis of all chi-square results obtained during the development of this research was performed, that is, results were verified for the four models presented previously, the results of the various tests developed to identify the respecified model, and a new programming developed in the simulated model increasing the number of interactions to 50, 100, and 200.

According to this analysis, it was possible to verify that the chi-square results obtained in the several tests developed in this research presented significant values, in agreement with the propositions established by Maccallum et al. [62]. The final simulated model has 18 degrees of freedom and when it considers the propositions established by Diamantopoulos and Siguaw [58], which consider a value of 0.80 enough for “more practical purposes.”

In the development of the structural model of this research, a sample of two hundred and eighty-eight responses (*N* = 288) was used, so there is a probability of detecting specification errors. However, the values of the chi-square statistic and the degrees of freedom for the various tests developed were considered, offering strong reasons to believe that there are no serious discrepancies between the hypothetical model and the data, that is, the data obtained from the survey applied to organizations accredited by the ONA Accreditation Methodology, and simulated data from these data fit the model that evaluates the sustainability of accredited organizations.

## 5. Discussion

The results presented in Figure 3, referring to the structural equations shown in Table 3, provide some valuable information about the impact of accreditation on the sustainability of health services. More specifically, it is possible to empirically recognize the importance of the five determinants of health service quality management, leadership (L), people management (PM), organizational culture (OC), process orientation (PO), and safety (S), directly affecting accreditation process and consequently organizational sustainability. Evaluating the leadership construct (standardized coefficient = 0.70), this relationship is the basis for a strong argument that the quality team, which is formed by leadership, is involved in quality improvement processes, considering accreditation as a fundamental tool. Evaluating the people management construct, which presents results with 77.0% of explanations (communalities), we can see that the hypothesis that tests its influence in people management presents positive results, however, with a low correlation value (0.22). This result is due to the fact that in Brazil the people management factor is still managed by a department that deals only with legal aspects, often having no involvement with quality management.

Values of positive and strong significance (standard coefficient = 0.86) are found between the relationships of organizational culture in quality management. In Brazil, the organizational culture is present and visible on a daily basis, involving all sectors of the company affecting directly the processes developed within the organization. This is one of the main difficulties for organizations in the beginning of the accreditation process. The influence between factor orientation for processes in quality management was confirmed starting from the standard coefficient = 0.46. This is due to the fact that organizations have been seeking greater productivity and quality in hospital services, combined with the enormous efforts to adapt to national, international, and service standards.

The related hypotheses between safety and quality management are also confirmed, however, with a standard coefficient = 0.19. In Brazil, the existence of processes related to safety is defined based on the administrative rule GM/MS Number 529/2013. The accredited health services work with the existence of patient safety cores, the obligation to report adverse events, and the elaboration of the patient safety plan. However, this factor is still perceived in isolation in many cases.

## 6. Conclusion

In this research, the hypothesized relationship between quality management and sustainability of the organizations accredited by the ONA methodology was empirically validated using an analytical trajectory model. From the obtained results, it was possible to conclude that the constructs, quality management, accreditation, sustainability, leadership, and organizational culture, show values of very strong positive significance for the model. That relationship confirms the importance of the constructs in hospital accreditation, in accordance with studies developed by several cited authors. Less strong positive estimates were identified in the relation of the constructs: people management, process orientation, and safety.

There are a number of directions in which this research can be extended. First, the interlinking of the internal measure of service quality that can be measured by hospital professionals as presented in this paper. The external measure of quality service can be explored together with customers.

In this case, the combination of survey methodology with structural equation modeling technique, tested in terms of dimensionality, reliability, convergent validity, and discriminant validity and validated from the leave-one-out method, can be used to prove the nature of relationships based on sustainability.

In management practice, the proposed model becomes a useful tool for managers at accredited companies providing knowledge of the effect of each construct over the organization results. So, managers can focus on their efforts, developing strategies to improve the factors that have contributed insufficiently, that is, that have low correlations in their relationships.

It was observed that the Brazilian accreditation methodology generates a positive impact on the accredited companies. With that, it is possible to better understand the influence of the constructs used in this work; the research provided a holistic view of existing processes in accredited organizations. Those have been working to increase the productivity and quality in hospital services, with combined efforts to meet national and international standards of provision of services.

The proposed model also provides an important instrument for measuring the factors that are directly related to the accreditation and sustainability of organizations accredited by ONA methodology and is a valuable tool not only for researchers, but also for managers, professionals, and accreditation's institutions, that allows the development of several actions, such as, people management, improvement of quality practices, and presence of a safety culture, as well as other factors that can affect the accreditation and sustainability.

Finally, the results of this empirical study provided a direction and trend of quality management in the health care industry. The proposed model proves that the Brazilian accreditation methodology has generated influence in the aspects related to the sustainability of accredited health organizations and that the formalization of a quality program, with a desire for accreditation, provides greater sustainability for the organizations.

## Figures and Tables

**Table 1:** Quality constructs used in hospitals.

Leadership (L)	Related to the commitment of the top management, traditionally considered one of the most powerful forces of quality management.	Xiong et al., 2015; Douglas et al., 2004; Meyer et al., 2001; Woo et al., 2013; Kunst and Lemmink, 2000; McFadden et al., 2015; El-jardali et al., 2008; Lee et al., 2013; Moon et al., 2008; Faye et al., 2013.
Sustainability (SY)	Related to the capacity of organizations to be able to withstand the challenges and variations over time, through a process of continuous improvement.	Slaghuis et al., 2013; Xiong et al., 2015; Li et al., 2002; Lee et al., 2013; Goldstein and Naor, 2005; Kunst and Lemmink, 2000.
People management construct (PM)	Describes how the organization engages, manages, and develops its workforce.	Choi et al., 2013; Douglas et al., 2004; Phichitchaisopa and Naenna, 2013; Lee et al., 2013; Li et al., 2003; Xiong et al., 2015; Mcfadden et al., 2015; Awuor and Kinuthia, 2013; Choi et al., 2013.
Organizational culture construct (O)	Refers to the “state” or set of characteristics that describes affective commitment and the desire to pursue a course in action with a focus on the target.	Nicolas et al., 2006; Woo et al., 2013; Awuor and Kinuthia, 2013; Cheng et al., 2014; Faye et al., 2013; El-Jardali et al. 2008; Woo et al. 2013.
Quality management construct (Q)	The literature indicates that the qualities in health organizations include quality practices related to customer satisfaction.	Xiong et al., 2015; El-Jardali et al., 2008; Douglas et al., 2004; Mcfadden et al., 2015; Awuor and Kinuthia, 2013; Choi et al., 2013; Cheng et al., 2014.
Process-oriented construct (P)	Process-oriented activities as related to the existence of well-defined processes in all activities of the organization.	Boyer et al., 2012; Kunkel et al., 2007; Kunst and Lemmink, 2000; Awuor and Kinuthia, 2013; Claver et al., 2003; Gowen et al., 2006; Kunkel et al., 2007; Lee et al., 2012.
Safety construct (S)	Refers to the common perceptions of an organization's members about their security policies and practices, which are directly influenced by top management.	Boyer et al., 2012; Mcfadden et al., 2015; Woo et al., 2013.
Accreditation construct (A)	Refers to a voluntary evaluation method that aims to guarantee the quality of health services through standards previously defined by ONA.	El-Jardali et al., 2008; Woo et al., 2013.

**Table 2:** Variables from (1).

y=y1y2⋮yp	(*p* × 1) vector of the *p*-dependent variables, or manifest response
$\Lambda_{y}=\left[\begin{array}{cccc} \lambda_{11} & \lambda_{12} & \vdots & \lambda_{1r}\\ \lambda_{21} & \lambda_{22} & \vdots & \lambda_{2r}\\ \vdots & \vdots & \vdots & \vdots \\ \lambda_{p1} & \lambda_{p2} & \vdots & \lambda_{pr} \end{array}\right]$	(*p* × *r*) matrix of the factorial weights of *η* in *y*
η=η1η2⋮ηr	(*r* × 1) vector of the *r*-dependent latent variables
ε=ε1ε2⋮εp	(*p* × 1) vector of the measurement errors of *y*

**Table 3:** Quality constructs used in hospitals.

Construct	Equation
Leadership	*Y*_1*k*_ = Λ_*ysek*_^∗^*η*_*l*_ + *ε*_*lk*_
Sustainability	*Y*_*sek*_ = Λ_*ysek*_^∗^*η*_*s*_ + *ε*_*sek*_
People management	Λ_*ygpk*_ = ∗*η*_*gp*_ + *ε*_*gpk*_
Organizational culture	*Y*_*ck*_ = Λ_*yck*_^∗^*η*_*gp*_ + *ε*_*gpk*_
Quality management	*Y*_*qk*_ = Λ_*yqk*_^∗^*η*_*q*_ + *ε*_*qk*_
Process orientation	*Y*_*pk*_ = Λ_*ypk*_^∗^*η*_*p*_ + *ε*_*pk*_
Safety	*Y*_*pk*_ = Λ_*ypk*_^∗^*η*_*p*_ + *ε*_*pk*_
Accreditation	*Y*_*ak*_ = Λ_*yak*_^∗^*η*_*a*_ + *ε*_*ak*_

*k* = 1, 2,…, 6.

**Table 4:** Relationships established between the constructs selected in the model.

Construct	Measurement items	Variable type	Direct influence from constructs	Indirect influence from constructs	Direct influence on constructs	Indirect influence on constructs
Leadership	6	Exogenous	—	—	Q	A, SY
People management	5	Exogenous	—	—	Q	A, SY
Organizational culture	4	Exogenous	—	—	Q	A, SY
Quality management	5	Endogenous	L, PM, O, P, S	—	A	SY
Process orientation	4	Exogenous	—	—	Q	A, SY
Safety	5	Exogenous	—	—	Q	A, SY
Accreditation	4	Endogenous	Q	L, PM, O, P, S	SY	—
Sustainability	6	Endogenous	A	Q, L, PM, O, P, S	—	—

**Table 5:** Correlation matrix for the constructs.

	F1L	F2L	F1SY	F2SY	F3SY	F1PM	F2PM	F1O	F2O	F1Q	F2Q	F1P	F1S	F2S	F1A
F1L	1.000	0.288	0.278	0.294	0.297	0.269	0.572	0.288	0.335	0.308	0.290	0.251	0.572	0.523	0.517
F2L	0.288	1.000	0.323	0.342	0.345	0.313	0.523	0.278	0.323	0.298	0.280	0.242	0.665	0.608	0.274
F1SY	0.278	0.323	1.000	0.315	0.317	0.288	0.288	0.294	0.342	0.315	0.296	0.257	0.612	0.560	0.446
F2SY	0.294	0.342	0.315	1.000	0.298	0.271	0.278	0.297	0.345	0.317	0.298	0.259	0.575	0.526	0.632
F3SY	0.297	0.345	0.317	0.298	1.000	0.235	0.294	0.269	0.313	0.288	0.271	0.235	0.499	0.456	0.575
F1PM	0.269	0.313	0.288	0.271	0.235	1.000	0.297	0.572	0.523	0.288	0.278	0.294	0.297	0.269	0.526
F2PM	0.572	0.523	0.288	0.278	0.294	0.297	1.000	0.665	0.608	0.335	0.323	0.342	0.345	0.313	0.290
F1O	0.288	0.278	0.294	0.297	0.269	0.572	0.665	1.000	0.560	0.308	0.298	0.315	0.317	0.288	0.280
F2O	0.335	0.323	0.342	0.345	0.313	0.523	0.608	0.560	1.000	0.290	0.280	0.296	0.298	0.271	0.296
F1Q	0.308	0.298	0.315	0.317	0.288	0.288	0.335	0.308	0.290	1.000	0.242	0.257	0.259	0.235	0.298
F2Q	0.290	0.280	0.296	0.298	0.271	0.278	0.323	0.298	0.280	0.242	1.000	0.269	0.313	0.288	0.271
F1P	0.251	0.242	0.257	0.259	0.235	0.294	0.342	0.315	0.296	0.257	0.269	1.000	0.857	0.654	0.554
F1S	0.572	0.665	0.612	0.575	0.499	0.297	0.345	0.317	0.298	0.259	0.313	0.857	1.000	0.488	1.868
F2S	0.523	0.608	0.560	0.526	0.456	0.269	0.313	0.288	0.271	0.235	0.288	0.654	0.488	1.000	0.456
F1A	0.517	0.274	0.446	0.632	0.575	0.526	0.290	0.280	0.296	0.298	0.271	0.554	1.868	0.456	1.000

**Table 6:** Factor analysis results.

Construct	Variable	Factor 1% var	Factor 2% var	Factor 3% var	Communalities % explanation
Leadership	F1—acting	0.379			75.4%
	F2—involvement		0.375		

Sustainability	F1—performance	0.297			78.5%
	F2—commitment		0.255		
	F3—goals			0.233	

People management	F1—information	0.645			77.0%
	F2—value		0.125		

Organizational culture	F1—commitment	0.570			81.4%
	F2—performance		0.244		

Quality management	F1—team involvement	0.437			76.9%
	F2—indicators		0.332		

Process orientation	F1—process orientation	0.820			82.0%
Safety	F1—safety culture	0.544			77.7%
	F2—risk		0.233		

Accreditation	F1—accreditation	0.865			86.5

**Table 7:** Model fit indices.

Type of indicator	Indicator	Result	Reference values
Absolute fit	*χ* ^2^—chi-square	57.25	*p* value > 0.05
	Degrees of freedom (df)	18	Greater than 1
	Normed chi-square	3.18	Between 1 and 3: good fit Greater than 5: bad fit
	Goodness-of-fit index (GFI)	0.977	≥0.90
	Root mean square residual (RMR)	0.319	≤0.05
	Standardized root mean residual (SRMR)	0.113	≥0.1

Incremental fit	Normed fit index (NFI)	0.949	≥0.9
	Comparative fit index (CFI)	0.967	≥0.9
Parsimonious fit	Adjusted goodness of fit index (AGFI)	0.894	≥0.9
	Parsimony normed fit index (PNFI)	0.735	Greater value: better fit
	Parsimony goodness-of-fit index (PGFI)	0.486	≤0.67; but 0.5 is a good fit

Populacional fit	Root mean square error of approximation (RMSEA)	0.470	Between 0.03 and 0.08; 0.05 is a good fit

## References

[1] Shaw C. D., Braithwaite J., Moldovan M. (2013). Profiling health-care accreditation organizations: an international survey. *International Journal for Quality in Health Care*.

[2] Lee D. (2012). Implementation of quality programs in health care organizations. *Service Business*.

[3] Claver E., Tarí J. J., Molina J. F. (2003). Critical factors and results of quality management: an empirical study. *Total Quality Management & Business Excellence*.

[4] Wiig S., Robert G., Anderson J. E. (2014). Applying different quality and safety models in healthcare improvement work: boundary objects and system thinking. *Reliability Engineering & System Safety*.

[5] Ashill N. J., Carruthers J., Krisjanous J. (2006). The effect of management commitment to service quality on frontline employees’ affective and performance outcomes : an empirical investigation of the New Zealand public healthcare sector. *International Journal of Nonprofit and Voluntary Sector Marketing*.

[6] Awuor E. O., Kinuthia D. M. (2013). Total quality management practices in selected private hospitals. *European Journal of Business and Management*.

[7] Chang S., Hsiao H., Huang L., Chang H. (2011). Taiwan quality indicator project and hospital productivity growth. *Omega*.

[8] Greenfield D., Braithwaite J. (2008). Health sector accreditation research: a systematic review. *International Journal for Quality in Health Care*.

[9] Groene O., Klazinga N., Wagner C. (2010). Investigating organizational quality improvement systems, patient empowerment, organizational culture, professional involvement and the quality of care in European hospitals: the “deepening our understanding of quality improvement in Europe (DUQuE)” project. *BMC Health Services Research*.

[10] Organização Nacional de Acreditação About ONA. https://www.ona.org.br/Pagina/20/A-ONA/.

[11] Schiesari L. M. C. (2014). Avaliação externa de organizações hospitalares no Brasil: podemos fazer diferente?. *Ciência & Saúde Coletiva*.

[12] Organização Nacional de Acreditação Organizações certificadas. https://www.ona.org.br/OrganizacoesCertificadas/.

[13] Short P. J., Rahim M. A. (1995). Total quality management in hospitals. *Total Quality Management*.

[14] Woldgebriel S., Kitaw D., Beshah B. (2014). Quality improvement approaches and models in healthcare. *Industrial Engineering & Management*.

[15] Society I., Care H. (2003). *Quality and Accreditation in Health Care Services: A Global Review Quality and Accreditation in Health Care*.

[16] Xiong J., He Z., Ke B., Zhang M. (2015). Development and validation of a measurement instrument for assessing quality management practices in hospitals: an exploratory study. *Total Quality Management & Business Excellence*.

[17] Robert G. B., Anderson J. E., Burnett S. J. (2011). A longitudinal, multi-level comparative study of quality and safety in European hospitals: the QUASER study protocol. *BMC Health Services Research*.

[18] Scheirer M. A. (2005). Is sustainability possible? A review and commentary on empirical studies of program sustainability. *American Journal of Evaluation*.

[19] Ahire S. L., Golhar D. Y., Waller M. A. (1996). Development and validation of TQM implementation constructs. *Decision Sciences*.

[20] Goldstein S. M., Naor M. (2005). Linking publicness to operations management practices: a study of quality management practices in hospitals. *Journal of Operations Management*.

[21] Lee K., Wan T. T. H., Kwon H. (2013). The relationship between healthcare information system and cost in hospital. *Personal and Ubiquitous Computing*.

[22] Lee S., Choi K. S., Kang H. Y., Cho W., Chae Y. M. I. (2002). Assessing the factors influencing continuous quality improvement implementation: experience in Korean hospitals. *International Journal for Quality in Health Care*.

[23] Meyer Goldstein S. M., Collier D. A. (2001). An empirical test of the causal relationships in the Baldridge health care pilot criteria. *Journal of Operations Management*.

[24] Kunst P., Lemmink J. (2000). Quality management and business performance in hospitals: a search for success parameters. *Total Quality Management*.

[25] Tejedor J. (2009). Reflexión sobre el fin último de la gestión en el sector hospitalario español. *Gaceta Sanitaria*.

[26] Donabedian A. (1988). The quality of care. How can it be assessed?. *JAMA*.

[27] Kunkel S., Rosenqvist U., Westerling R. (2007). The structure of quality systems is important to the process and outcome, an empirical study of 386 hospital departments in Sweden. *BMC Health Services Research*.

[28] Slaghuis S. S., Strating M. M. H., Bal R. A., Nieboer A. P. (2013). A measurement instrument for spread of quality improvement in healthcare. *International Journal for Quality in Health Care*.

[29] Douglas T. J., Fredendall L. D. (2004). Evaluating the deming management model of total quality in services. *Decision Sciences*.

[30] Woo J. S., Kim Y. H., Yoon B. J. (2013). The effects of accreditation program to the leadership, organizational culture, hospital management activities and performances - focused on perception of accredited hospital professions. *Korean Journal of Hospital Management*.

[31] Mcfadden K. L., Stock G. N., Gowen C. R. (2015). Leadership, safety climate, and continuous quality improvement. *Health Care Management Review*.

[32] El-Jardali F., Jamal D., Dimassi H., Ammar W., Tchaghchaghian V. (2008). The impact of hospital accreditation on quality of care: perception of Lebanese nurses. *International Journal for Quality in Health Care*.

[33] Moon J. Y., Lee S. C., Lee D. K., Suh Y. H. (2009). Analysis of causal relationship among performance factors of quality management in Korean public enterprises: using Malcolm Baldrige non-profit criteria. *Journal of the Korean Society for Quality Management*.

[34] Faye A., Fournier P., Diop I., Philibert A., Morestin F., Dumont A. (2013). Developing a tool to measure satisfaction among health professionals in sub-Saharan Africa. *Human Resources for Health*.

[35] Li L. X., Benton W. C., Leong G. K. (2002). The impact of strategic operations management decisions on community hospital performance. *Journal of Operations Management*.

[36] Choi W., Rho M. J., Park J., Kim K. J., Kwon Y. D., Choi I. Y. (2013). Information system success model for customer relationship management system in health promotion centers. *Healthcare Informatics Research*.

[37] Phichitchaisopa N., Naenna T. (2013). Factors affecting the adoption of healthcare information technology. *EXCLI Journal*.

[38] Li L., Benton W. (2003). Hospital capacity management decisions: emphasis on cost control and quality enhancement. *European Journal of Operational Research*.

[39] Nicolas B., Wilkinson T., Snell S. (2006). *Human Reseach Management: The Sage Handbook*.

[40] Cheng Y. M. (2014). Extending the expectation-confirmation model with quality and flow to explore nurses’ continued blended e-learning intention. *Information Technology & People*.

[41] Gowen C. R., Mcfadden K. L., Hoobler J. M., Tallon W. J. (2006). Exploring the efficacy of healthcare quality practices, employee commitment, and employee control. *Journal of Operations Management*.

[42] Lee S. M., Lee D., Olson D. L. (2012). Health-care quality management using the MBHCP excellence model. *Total Quality Management & Business Excellence*.

[43] Forza C. (2002). Survey research in operations management: a process-based perspective. *International Journal of Operations & Production Management*.

[44] Hair J. F., Anderson R. E., Babin B. J., Black W. C. (2010). Multivariate data analysis: overview of multivariate methods.

[45] Malhotra N. K. (1996). *Marketing Research: An Applied Orientation*.

[46] Bollen K. (1989). Structural equations with latent variables. *Sociological Methods and Research*.

[47] Marôco J. (2014). *Análise de Equações Estruturais: fundamentos teóricos, software & aplicações*.

[48] Ahire S. L., Devaraj S. (2001). An empirical comparison of statistical construct validation approaches. *IEEE Transactions on Engineering Management*.

[49] Kline R. B. (2011). *Principles and Practice of Structural Equation Modeling*.

[50] Reinartz W., Haenlein M., Henseler J. (2009). An empirical comparison of the efficacy of covariance-based and variance-based SEM. *International Journal of Research in Marketing*.

[51] Hill M. M., Hill A. (2008). *Investigação por questionário*.

[52] Alwin D. F. (2007). *Margins of Error: A Study of Reliability in Survey Measurements*.

[53] Anderson J., Gerbing D. (1988). Structural equation modeling in practice: a review and recommended two-step approach. *Psychological Bulletin*.

[54] Vieira A. L. (2011). *Interactive LISREL in Practice: Getting Started with a SIMPLIS Approach*.

[55] Baumgartner H., Homburg C. (1996). Applications of structural equation modeling in marketing and consumer research: a review. *International Journal of Research in Marketing*.

[56] Ping R. A. (2004). On assuring valid measures for theoretical models using survey data. *Journal of Business Research*.

[57] Brown T. A. (2006). *Confirmatory Factor Analysis for Applied Research*.

[58] Diamantopoulos A., Siguaw J. A. (2000). *Introducing LISREL*.

[59] Bentler P. M., Bonett D. G. (1980). Significance tests and goodness of fit in the analysis of covariance structures. *Psychological Bulletin*.

[60] Steenkamp J. B. E., Van Trijp H. C. (1991). The use of LISREL in validating marketing constructs. *International Journal of Research in Marketing*.

[61] Fornell C., Larcker D. F. (1981). Evaluating structural equation models with unobservable variables and measurement error. *Journal of Marketing Research*.

[62] Maccallum R. C., Browne M. W., Sugawara H. M. (1996). Power analysis and determination of sample size for covariance structure modeling of fit involving a particular measure of model. *Psychological Methods*.

